# In vitro and in vivo synergistic effects of cyclizine and piroxicam in combination with linezolid against methicillin-resistant *Staphylococcus aureus*

**DOI:** 10.1007/s00253-026-13738-9

**Published:** 2026-03-19

**Authors:** Mai A. Moawad, Abeer M. Abd El-Aziz, Mona I. Shaaban

**Affiliations:** Department of Microbiology and Immunology, Faculty of Pharmacy, Mansoura University, Mansoura, 35516 Egypt

**Keywords:** MRSA, LRSA, Linezolid, Cyclizine, Piroxicam, Synergistic, Bactericidal, Mouse model

## Abstract

**Abstract:**

**Background:**

Linezolid (LNZ) is considered one of the last-resort antimicrobial agents reserved for treating methicillin resistant *Staphylococcus aureus* (MRSA) and vancomycin-resistant *Staphylococcus aureus* (VRSA). The development of resistance against linezolid necessitates the exploration of novel therapies.  Aim: This study aims to investigate the synergistic activity of various combinations of linezolid with non-antibiotic through in vitro and in vivo approaches.

**Methods and Results:**

In our research, 44 *S. aureus* isolates were obtained from various clinical sources. *S. aureus* isolates presented high levels of resistance to β-lactams and moderate resistance to doxycycline and erythromycin. Among the isolates, 43 (97.73%) were MRSA, 10 (22.73%) were linezolid resistant *S. aureus* (LRSA), and 17 (38.64%) were classified as VRSA. A total of 97.73% of the isolates presented the *mecA* gene (MRSA), whereas the *optrA* gene was detected in 9.09% of the isolates (LRSA).  The synergistic activity of nine compounds with linezolid was assessed in vitro against LRSA isolates using broth microdilution and checkerboard microdilution methods. Linezolid/cyclizine and linezolid/piroxicam combinations showed fractional inhibitory concentration indexes (FICIs) ranging from 0.28 to 0.5 against LRSA isolates. Time-kill curves were used to confirm their bactericidal activity. Promising combinations (linezolid/cyclizine and linezolid/piroxicam) were further evaluated in vivo LRSA-induced lung infection murine animal model. Compared with monotherapy, combination therapies significantly enhance bacterial eradication and increase sensitivity to linezolid, resulting in superior bacterial eradication. Linezolid/cyclizine and linezolid/piroxicam combinations provided complete protection (100% survival), improved lung pathology, and enhanced clinical scores.

**Conclusion:**

This study presents two novel combination therapies (linezolid/cyclizine and linezolid/piroxicam) with promising applications in treating severe LRSA infections.

**Key points:**

- *optrA gene was detected in four linezolid S. aureus–resistant isolates (LRSA)*
- *Linezolid/cyclizine and linezolid/piroxicam synergism was detected against LRSA.*
- *Combinations revealed complete lung protection in lung-infected LRSA murine model.*

**Supplementary Information:**

The online version contains supplementary material available at 10.1007/s00253-026-13738-9.

## Introduction

*S. aureus* is a dangerous microorganism that causes infections in both community and healthcare settings. It can colonize healthy individuals without causing symptoms (Chambers and Deleo 2009). *S. aureus* is responsible for various infections like bacteremia, endocarditis, osteoarticular infections, and skin infections (Paling et al. 2017). It can also lead to conditions like abscesses, meningitis, toxic shock syndrome, and urinary tract infections (Gajdács and Urbán 2019). The pathogenicity of *S. aureus* is attributed to its numerous virulence factors that impact the immune system (Tam and Torres 2019).

The rise of antibiotic-resistant bacterial pathogens, particularly gram-positive organisms like MRSA, poses a significant challenge for clinicians and microbiologists. The decreasing effectiveness of vancomycin and linezolid against MRSA strains highlights the necessity of new therapeutic options for clinical practice (Bayer et al. 2013).

Linezolid is a synthetic oxazolidinone antibiotic with a unique chemical structure and mechanism of action (Jorgensen et al. 1997). It is highly effective against multidrug-resistant gram-positive pathogens, including vancomycin-resistant *Enterococcus faecalis* and *Enterococcus faecium*, penicillin-resistant *Streptococcus pneumoniae*, and MRSA strains (Belousoff, Eyal et al. 2017). Linezolid inhibits bacterial protein synthesis by binding to the catalytic site on the 50S ribosomal subunit, specifically at the 12′ ribosomal peptidyl transferase center (PTC), preventing tRNA from binding to the ribosomal A-site and blocking peptide bond formation, thereby inhibiting protein synthesis (Vázquez-Laslop and Mankin 2018). Resistance to linezolid in *S. aureus* was reported in China in the first year of its clinical use (Shariati et al. 2020; Han et al. 2022). The increasing prevalence of linezolid-resistant MRSA (LR-MRSA) worldwide is a cause for concern.

Combination therapy with antimicrobial agents can provide broad-spectrum coverage, prevent resistance, and enhance effectiveness. Combining linezolid with other antimicrobial agents is recommended due to the potential limitations of linezolid monotherapy. Innovative techniques are needed to address the challenge of antimicrobial resistance. Previous studies have explored the effectiveness of linezolid in combination with various antimicrobial agents (Grif et al. 2001; Grohs, Kitzis et al. 2003). However, linezolid combination with daptomycin, vancomycin (Luther, LaPlante et al. 2015), or ciprofloxacin (Grohs, Kitzis et al. 2003) exhibited antagonistic effects against MRSA. Repurposing non-antimicrobial, FDA-approved drugs in combination with antibiotics is a promising strategy to treat multi-drug-resistant (MDR) infections and combat antibiotic resistance (Jacqueline et al. 2005). By using drugs with known safety profiles, this method accelerates the development of new treatments while avoiding the long, costly process of de novo drug discovery (Tiwana, Cock et al. 2025). However, there is a lack of research on the effectiveness of linezolid combinations with approved medications other than antimicrobial agents. So, we applied this strategy by selecting nine FDA-approved compounds that have been reported to exhibit antimicrobial activity. The drugs have no adverse effects and are not contraindicated with linezolid. Cyclizine demonstrated antimycobacterial activity against *Mycobacterium* *abscessus* strains (Kirkwood et al. 2018). A complex synthesized with piroxicam showed activity against gram-positive cocci (Marinescu, Culita et al. 2016). Vitamin B12 exhibited synergistic activity with linezolid against MRSA (Shahzad, Ashraf et al. 2018). Additionally, carbonyl cyanide m-chlorophenylhydrazone (CCCP) (Lu et al. 2020; Chen, Teng et al. 2021), N-acetyl cysteine (Hamed et al., 2022), ondansetron (Zou, Zhou et al. 2017), hyoscine-n-butyl bromide (Mohamed, Abd El-Baky et al. 2024), dexamethasone (O’Neil et al. 2023), and l-carnitine (Hassan,Abdel-Monem et al. 2024) also showed antimicrobial activity.

Hence, our study seeks to ascertain the prevalence of linezolid resistance among *S. aureus* isolates sourced from diverse clinical specimens. Additionally, we evaluated FDA-endorsed drugs for their potential synergistic interactions with linezolid. Synergistic agents that augmented the effectiveness of linezolid were assessed through the checkerboard microdilution and time–kill assay methods. The combined efficacy was further confirmed using an in vivo animal model.

## Methods

### Bacterial isolation and identification

Forty-eight isolates were collected from various clinical sources over the period from August 2020 to December 2021, including wounds, sputum, blood, eye swabs, nasal discharge, urine, and diabetic foot ulcers, from different hospitals of Mansoura University, Al-Qasr Al-Aini Hospitals, Tanta University, Kafr El-Sheikh University, and Cairo University, Egypt. All the isolates were obtained in accordance with the approval of the Research Ethics Committee, Faculty of Pharmacy, Mansoura University, with ethical codes 2020−67.

Isolates were collected and purified using standard microbiological techniques (Koneman and P. 1997). Under the microscope, *S. aureus* was detected as gram-positive cocci organized in grape-like clusters. Mannitol salt agar media (Oxoid, Thermo Fisher, Basingstoke, UK) were used to selectively purify the *S. aureus* isolates. Furthermore, all the isolates were subjected to coagulase and catalase production tests (Boerlin et al. 2003).

The inclusion criteria for selecting the 48 staphylococcal isolates were being collected from moderate to severe human clinical infections and identified as a pure single strain per patient through microbiological investigations. Isolates from patients with incomplete data, or those who had received more than 72 h of systemic antibiotics before sampling, were excluded.

### Antimicrobial susceptibility testing and phenotypic identification of MRSA

The Kirby–Bauer disc diffusion method was used to test the antimicrobial susceptibility of all the identified isolates following the Clinical Laboratory Standard Institute (CLSI) guidelines (CLSI 2021). Cultures were swabbed onto sterile Mueller–Hinton agar plates (Oxoid, Thermo Fisher, Basingstoke, UK) after the pure bacterial cultures were diluted to 0.5 McFarland standard. Ten antimicrobial discs (Oxoid, Thermo Fisher, Basingstoke, UK) were applied, including amoxicillin/clavulanic acid (AMC, 20/10 μg), cefoxitin (FOX, 30 μg), cefotaxime (CTX, 30 μg), imipenem (IPM, 10 μg), gentamicin (CN, 30 μg), doxycycline (DO, 30 μg), levofloxacin (LEV, 5 μg), erythromycin (E, 15 μg), clindamycin (DA, 2 μg), and linezolid (LNZ, 30 μg). The diameter of the inhibition zone of each disc was measured after the plates were incubated at 37 °C, and the results were interpreted according to CLSI (CLSI 2021). MRSA isolates were identified by testing their susceptibility to FOX (30 μg) using the disc diffusion method, and the *mecA* gene detection using PCR. LRSA isolates were identified using the disc diffusion method by testing their susceptibility to LNZ (30 μg) according to CLSI (CLSI 2021).

### Determination of minimum inhibitory concentration (MIC) of vancomycin

The broth microdilution method was used to determine the vancomycin MIC for *S. aureus* isolates using 96-well microtiter plates with Mueller–Hinton broth (Oxoid, Thermo Fisher, Basingstoke, UK) (CLSI 2021). A concentration of 4096 μg/ml was prepared for vancomycin (Mylan, Egypt), followed by the preparation of two-fold serial dilutions in the subsequent wells. Overnight cultures of the tested *S. aureus* isolate were adjusted to 1 × 10^6^ CFU/ml, and 10 µl of each adjusted bacterial culture mixture was added to each well. After overnight incubation, the MIC of vancomycin was determined by identifying the lowest concentration that prevented visible growth of the tested isolates (Shady, El-Essawy et al. 2012). The susceptibility of *S. aureus* isolates to vancomycin was defined using the MIC interpretive criteria from CLSI 2021.

### Molecular detection of *S. aureus*–resistant isolates and DNA extraction

Colony PCR was carried out by suspending 1–2 fresh pure colonies into 100 μl of sterile nuclease-free water (Thermo Fisher Scientific, Waltham, MA, USA) into a 0.2-ml sterile PCR tube. The rapid DNA extraction method was performed according to Zhang et al. (Zhang et al. 2004). The culture suspension was heated at 95 °C for 10 min and then centrifuged at 5000 rpm for 5 min. The final DNA-containing supernatant was stored at −20 °C.

### Polymerase chain reaction (PCR) for *S. aureus* isolates

Amplification of the *mecA* gene using PCR was performed to detect methicillin resistance (MRSA), and the amplification of *cfr*, *cfr(B)*, and *optrA* genes was performed for the detection of linezolid-resistant *S. aureus* (LRSA). These genes were amplified using Dream Taq Green PCR Master Mix (2X) according to the manufacturer’s instructions (Thermo Fisher Scientific, Waltham, MA, USA), supplementary Table 5. PCR amplicons were detected using agarose gel 2% w/v agarose gel stained with ethidium bromide and compared with a 100-base plus DNA marker (Thermo Fisher Scientific, Waltham, MA, USA).

### Effect of different compounds on linezolid-resistant isolates

Nine different compounds were tested, including an antihistaminic and antiemetic drug (cyclizine) (CYC) (AMOUN, Egypt), a nonsteroidal anti-inflammatory drug (NSAID) (piroxicam) (PIR) (PFIZER, USA), a vitamin (vitamin B12 in the form of cyanocobalamin) (B) (MISR, Egypt), an efflux inhibitor (carbonyl cyanide m-chlorophenyl hydrazine) (CCCP) (Sigma-Aldrich, St. Louis, MO, USA), an antidote of acetaminophen (N-acetyl cysteine) (NAC) (Zambon, Switzerland), an antiemetic drug (ondansetron) (OND) (ADWIA, Egypt), an antispasmodic (hyoscine-n-butyl bromide) (HBB) (SANOFI), a corticosteroid (dexamethasone) (DEX) (EPICO,Egypt), and an antioxidant (l-carnitine) (LC) (Mepaco, Egypt), as potential inhibitors of linezolid resistance.

The linezolid-resistant isolates (no. 22, 35, 40, and 41) positive for the *optrA* gene were selected. The MIC of the tested compounds was determined using the broth microdilution method against selected *S. aureus* isolates (CLSI 2021). The MIC of linezolid was detected alone and in combination with sub-MIC of each of the tested compounds. *S. aureus* growth was detected by adding 40 μl of TTC (Sigma-Aldrich, St. Louis, MO, USA) (100 μg/ml) to visualize the red color (Mahfouz et al. 2023).

### Checkerboard microdilution method

The synergistic effects of the nine tested compounds on linezolid resistance were tested against four selected *S. aureus* isolates (no. 22, 35, 40, and 41). Five promising drugs (cyclizine, piroxicam, CCCP, vitB12, and N-acetyl cysteine) synergistically inhibited linezolid resistance using the checkerboard microdilution method against selected *S. aureus* isolates. Five combination groups were prepared, including linezolid plus five promising potential inhibitors (cyclizine, piroxicam, CCCP, vitB12, and N-acetyl cysteine).

Briefly, in sterile tubes, two-fold serial dilutions of linezolid (1 to 1024 μg/ml) and cyclizine (146.68 to 9387.5 μg/ml), piroxicam (78.13 to 10,000 μg/ml), CCCP (3.13 to 400 μg/mL), vitB12 (7.81 to 1000 μg/ml), and N-acetyl cysteine (100,000 to 781.25 μg/ml)) were prepared. A concentration of 1/16 to fourfold the estimated MIC of each tested compound was prepared in the checkerboard. In a 96-well microtiter plate, 50 μl of the diluted potential inhibitor was added to 50 μl of linezolid, resulting in a total volume of 100 μl in each well. Each well was inoculated with 10 μl of the diluted culture (5 × 10^6^ CFU/ml). Positive and negative controls were prepared for each combination, and the plates were incubated for 24 h at 37 °C. MICs were determined for linezolid and potential inhibitors alone and in combination by adding 40 μl of the TTC solution (100 μg/ml) to each well. The fractional inhibitory concentration (FIC) was determined for each combination to determine the type of interaction (Hall, Middleton et al. 1983; Hsieh, Chen et al. 1993).

### Time–kill assay

For each isolate, linezolid was tested at 1× MIC and 2× MIC alone and in combination with synergistic compounds (cyclizine and piroxicam) at a concentration of 0.25× MIC. MH broth with an inoculum of 5 × 10^4^ to 1 × 10^5^ CFU/ml were used in time‒kill curve studies in the presence of linezolid alone or the combination of both linezolid and the synergistic compound (Pearson et al. 1980). A positive control flask was also prepared using MH broth with no antibiotics.

An aliquot was taken from each flask at each time point (0, 2, 4, 6, 8, and 24 h), consisting of 100 μl taken from the flask and diluted with 900 μl of sterile saline. The initial volume of each flask was then restored with MH broth to its original volume to prevent any drug carryover. The surface drop method was used to calculate colony-forming unit (CFU). All experiments were conducted in triplicate, and the means were calculated (Pearson et al. 1980). The time–kill experiments were evaluated at various time intervals (0, 2, 4, 6, 8, and 24 h). The bactericidal effect was detected as a ≥ 3-log_10_ CFU/ml decrease, the synergistic effect as a ≥ 2-log_10_ CFU/ml decrease for a tested combination compared with the most active single compound, the additive effect as a 1 to 2-log_10_ CFU/ml decrease in the combination final colony count compared with the most active single compound, and the antagonistic effect as an increase to ≥ 1-log_10_ CFU/ml for the combination compared with the least active single compound (Lim et al. 2015; Gómez-Junyent et al. 2019).

### In vivo assay

#### Ethical statement

The ethical guidelines for research involving laboratory animals were followed to carry out all the animal procedures involved in our study. These guidelines were approved by the Ethical Committee of the Faculty of Pharmacy, Mansoura University, Egypt, following the Principles of Laboratory Animal Care (National Institutes of Health publication 85-23, revised 1985) (ethical approval code 2020-67).

#### Mice

Six-week-old female BALB-C mice, weighing 25 to 30 g and provided with food and water, were purchased from the Department of Pharmacology and Toxicology, Faculty of Pharmacy, Mansoura University (Bubeck Wardenburg et al. 2007).

The murine pneumonia model is frequently used to study the synergistic activity of linezolid with other antimicrobial agents against MRSA infections since linezolid exhibits excellent pulmonary penetration, making this model highly suitable for studying synergy in lungs (Zhou, Xiong et al. 2018). Additionally, the standardized mouse pneumonia model has been successfully used for studying gram-positive bacteria (Vera-Yunca, Matias et al. 2025).

#### Dose selection

Seven groups of mice (10 each) were lightly anesthetized with 2.5% (v/v) fluothane (AstraZeneca), and 50 μl of saline infected with 10^9^ CFU/ml linezolid-resistant *S. aureus* isolate no. 40 (LRSA40) was administered into the mouse nostrils. In addition, a negative control group (uninfected group) received saline only without infection or treatment (Bubeck Wardenburg et al. 2007).

After 16 h (the first dose of treatment) to 24 h (the second dose of treatment) after infection, the mice received different treatments: the infected group received saline intranasally, the CYC group received cyclizine (1.41 mg/mouse) intraperitoneally, the PIR group received piroxicam (0.04 mg/mouse) intranasally, and the LNZ group received linezolid (0.1 mg/mouse) intranasally. The remaining two groups received combinations of treatments: linezolid (0.1 mg/mouse) intranasally with cyclizine (1.41 mg/mouse) intraperitoneally and linezolid (0.1 mg/mouse) intranasally with piroxicam (0.04 mg/mice) (Supplementary Table 6). Signs of disease progression during the experiment were defined and scored for 7 days for each mice group, as previously described (Jacqueline et al. 2005; Mook-Kanamori et al. 2012; Ali et al. 2014). At specified time intervals, the mice were lightly anesthetized, and blood samples were obtained through eye puncture, followed by immediate euthanasia through cervical dislocation. Under aseptic conditions, the lungs were collected and then homogenized. Blood and lung viable bacterial counts were determined by the surface drop microdilution method using mannitol salt agar as selective media (DeMaria and Kapral 1978; Ali et al. 2014).

#### Statistical analysis of the results

The mean and standard deviation for each experiment, which was conducted in triplicate, were determined using an Excel spreadsheet. GraphPad Prism software (version 9.00) was utilized for the statistical analyses of our data. Statistical analysis for comparisons between two groups where one group represents the effect before adding a potential inhibitor and the other group indicates the effect after its addition was conducted via two-tailed paired *t*-tests. The results were considered statistically significant when the probability values (*P* values) were < 0.05.

## Results

### Isolation and identification of isolates

**Fig. 1:**
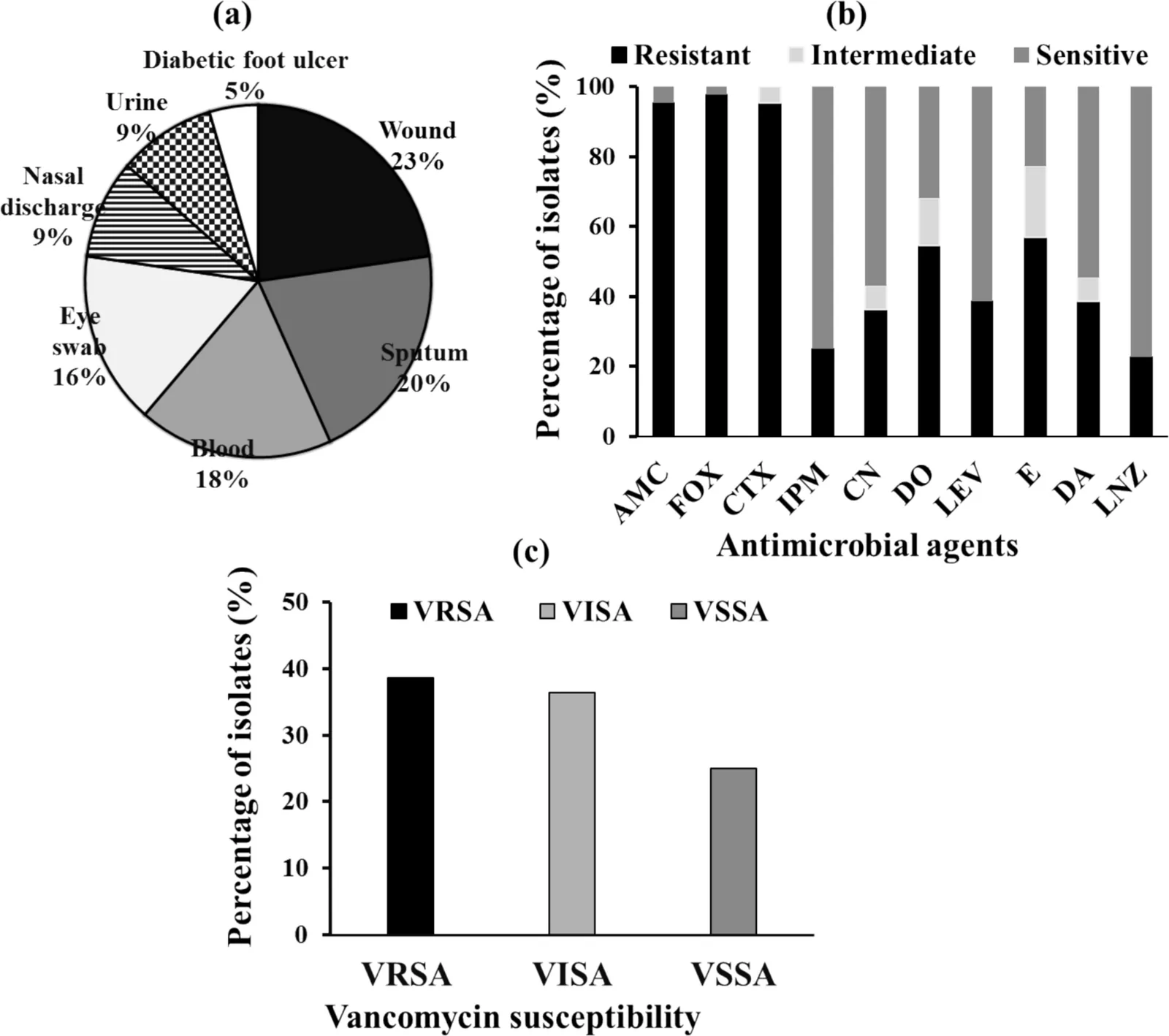
Clinical sources and antimicrobial resistance of the tested isolates. **a** Prevalence of *S. aureus* among different clinical sources. **b** Prevalence of antimicrobial resistance among *S. aureus* isolates. **c** Prevalence of vancomycin susceptibility among *S. aureus* isolates. AMC, amoxicillin-clavulanic acid; FOX, cefoxitin; CTX, cefotaxime; IPM, imipenem; CN, gentamicin; DO, doxycycline; LEV, levofloxacin; E, erythromycin; DA, clindamycin; LNZ, linezolid. VRSA, vancomycin-resistant *S. aureus*; VISA, vancomycin-intermediate *S. aureus*; VSSA, vancomycin-sensitive *S. aureus*

In this study, forty-eight staphylococcal samples were obtained from various hospitals in Egypt. On the basis of microscopic examination and biochemical reactions, forty-four (91.67%) isolates were identified as *S. aureus* (*n* = 1–44). These isolates were collected from different clinical sources (Supplementary Table 1), with the highest percentages coming from wounds (23%), followed by sputum (20%), blood ( 18%), eye swabs (16%), nasal discharge (9%), and urine (9%). The least common type of specimen was diabetic foot ulcers ( 5%) (Fig. 1a).

### Antimicrobial susceptibility testing

The susceptibility of 44 clinical isolates of *S. aureus* to various classes of antimicrobial agents was determined using the Kirby–Bauer disk diffusion method according to breakpoints indicated in CLSI guidelines (CLSI 2021). The isolates displayed varying levels of resistance. The antimicrobial susceptibility test demonstrated a high level of resistance to β-lactams, such as amoxicillin-clavulanic acid (95.45%), cefoxitin (97.73%), and cefotaxime (95.45%). The resistance levels were moderate toward tetracyclines, including doxycycline (54.55%); macrolides, such as erythromycin (56.82%); and lincosamides, such as clindamycin (38.64%). Linezolid (77.27%), imipenem (75%), levofloxacin (61.36%), and gentamicin (56.82%) were the most effective antibiotics for the management of infections caused by *S. aureus* (Fig. 1b and supplementary Table 1). Among the isolates, 43 (97.73%) were identified as MRSA because of their resistance to cefoxitin, while 10 isolates (22.73%) were identified as LRSA because of their resistance to linezolid.

### Vancomycin susceptibility of MRSA isolates

Vancomycin minimum inhibitory concentrations (MICs) against all *S. aureus* isolates were determined using the broth microdilution method. The results revealed that among the 44 *S. aureus* isolates, 17 (38.64%) exhibited MICs ≥ 16 μg/ml and were considered vancomycin resistant. Moreover, 16 isolates (36.36%) exhibited MIC values ranging from 4 to 8 μg/ml and were considered vancomycin intermediates. The remaining 11 isolates (25%) had MICs < 2 μg/ml and were considered vancomycin-sensitive (Fig. 1c and supplementary Table 2).

### Molecular detection of methicillin and linezolid resistance genes

**Fig. 2:**
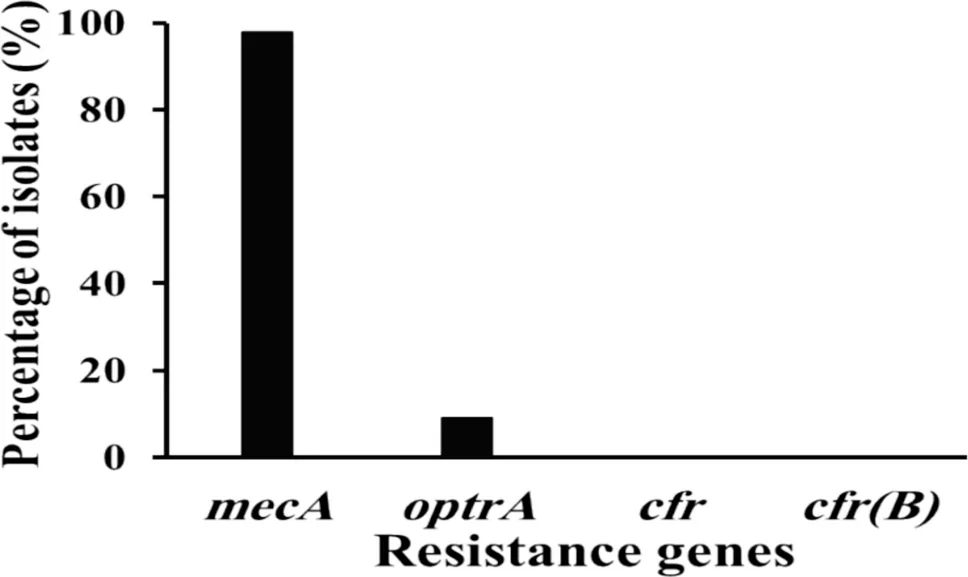
Molecular detection of different resistance genes among *S. aureus *isolates. Methicillin resistance gene (*mecA*) and linezolid resistance genes (*optrA*, *cfr*, and *cfr(B*))

Using PCR, the methicillin resistance gene (*mecA*) and linezolid resistance genes (*optrA*, *cfr*, and *cfr(B*)) were screened among 44 *S. aureus* isolates. The results revealed that 43 (97.73%) MRSA isolates presented the *mecA* gene, which was the most frequently detected gene (Supplementary Fig. 1a–d). On the other hand, PCR analysis revealed that the *optrA* gene was detected in only four (9.09%) resistant isolates (Supplementary Fig. 2), no. 22, 35, 40, and 41, while the *cfr* and *cfr(B)* genes were not detected (Fig. 2 and supplementary Table 3).

### In vitro synergistic evaluation of different compounds with linezolid

**Fig. 3:**
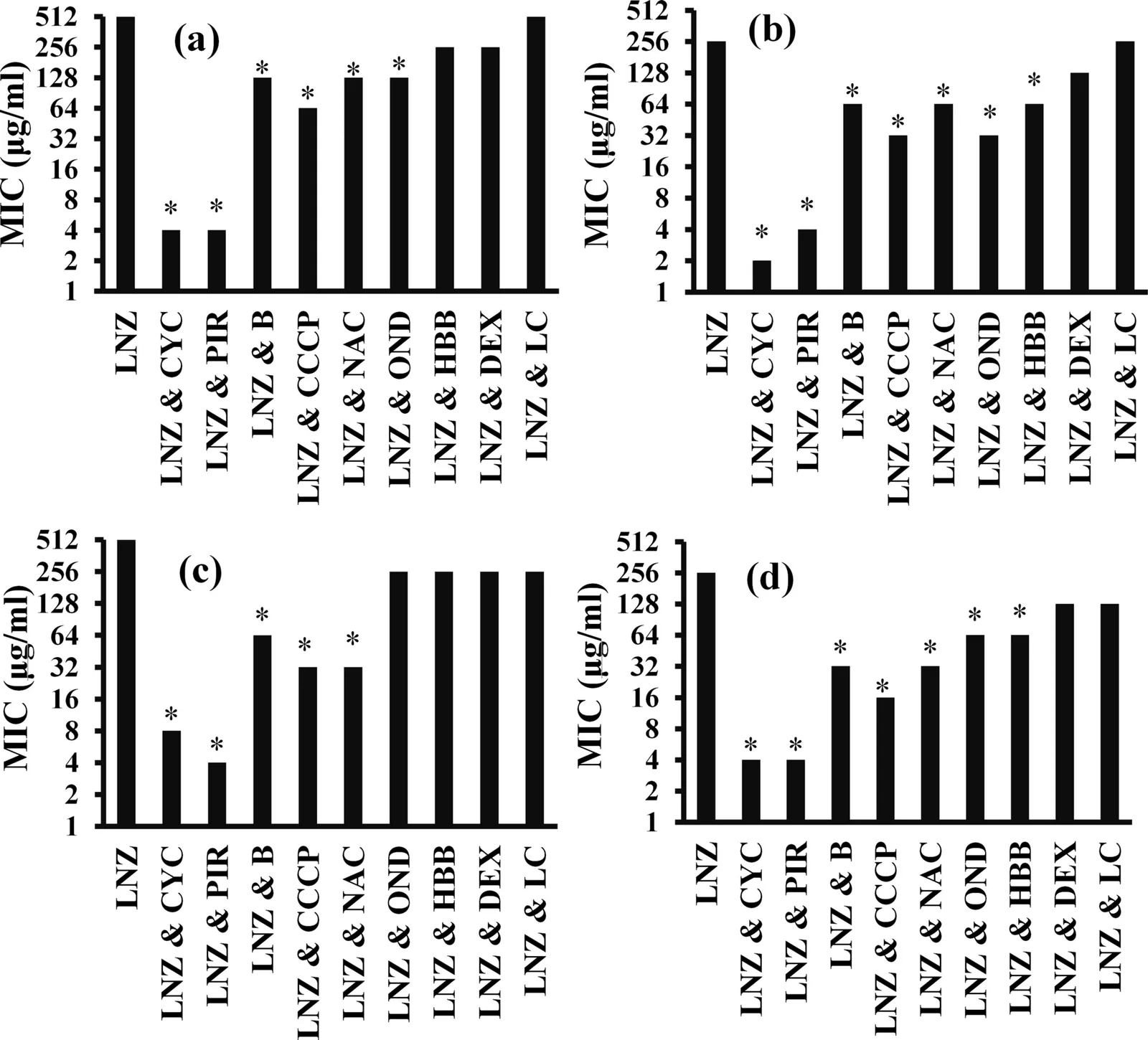
The effect of tested compounds on the MICs of linezolid using the broth microdilution method. **a** Isolate no. 22. **b** Isolate no. 35. **c** Isolate no. 40. **d** Isolate no. 41. MICs, minimum inhibitory concentrations; LNZ, linezolid; CYC, cyclizine; PIR, piroxicam; B, vitB12; CCCP, carbonyl cyanide m-chlorophenylhydrazine; NAC, N-acetyl cysteine; OND, ondansetron; HBB, hyoscine-n-butyl bromide; DEX, dexamethasone; LC, l-carnitine; *probability value (*P* value) is < 0.05, which is considered statistically significant

Various FDA-approved compounds were examined to determine their synergistic activity with linezolid against LR-MRSA isolates. Numerous in vitro and in vivo studies have been conducted to evaluate various compounds for their synergistic effects on linezolid (Jacqueline et al. 2005). The potential synergistic effects of sub-MICs of nine FDA-approved compounds (cyclizine, piroxicam, vitB12, CCCP, N-acetyl cysteine, ondansetron, hyoscine-n-butyl bromide, dexamethasone, and l-carnitine) combined with linezolid were initially assessed by the broth microdilution method against four LRSA isolates (no. 22, 35, 40, and 41). In the linezolid with cyclizine combination (LNZ + CYC), cyclizine significantly reduced the MICs of linezolid from 256–512 to 2–8 μg/ml. In addition, the combination of linezolid with piroxicam (LNZ + PIR), piroxicam reduced the MIC of linezolid to 4 μg/ml. VitB12, CCCP, N-acetyl cysteine, and ondansetron reduced the linezolid MIC by 2–16-fold for the four selected isolates. The remaining tested compounds (hyoscine-n-butyl bromide, dexamethasone, and l-carnitine) exhibited only a two-fold decrease in the linezolid MIC, whereas L-carnitine did not have any effect on linezolid against isolate no. 22 and 35. The best combination corresponded to linezolid with cyclizine and linezolid with piroxicam, which reduced the linezolid MIC by 64- to 128-fold. Moreover, linezolid combined with CCCP or vitB12 decreased the MIC of linezolid by 4- to 16-fold. Therefore, cyclizine, piroxicam, CCCP, vitB12, and N-acetyl cysteine were chosen for further experiments (Fig. 3 and supplementary Table 4).

### Checkerboard microdilution method

The checkerboard microdilution method was utilized to further assess the synergistic activity of various compounds in combination with linezolid. In our study, we calculated the minimum fractional inhibitory concentration index (FICI) for each combination, along with the concentrations of linezolid and the compounds at synergistic points. The most effective combinations were linezolid plus cyclizine, linezolid plus piroxicam, and linezolid plus CCCP, which had synergistic effects, with FICI values ranging from 0.28 to 0.5 against the four LRSA isolates. In contrast, linezolid combined with vitamin B12 and N-acetyl cysteine had less favorable outcomes, displaying an additive effect with FICI values between 0.63 and 1, respectively (Supplementary Figs. 3–4, Table 1).

**Table 1:** Checkerboard results for the combinations of linezolid with several compounds against the linezolid-resistant isolates

Isolate no.	FICI (interpretation) MICS (μg/ml) at synergistic point				
	LNZ/CYC	LNZ/PIR	LNZ/CCCP	LNZ/B	LNZ/NAC
22	0.5 (S)	0.38 (S)	0.5 (S)	1 (A)	1 (A)
35	0.38 (S)	0.5 (S)	0.38 (S)	0.75 (A)	1 (A)
40	0.25 (S)	0.38 (S)	0.38 (S)	0.63 (A)	0.75 (A)
41	0.31 (S)	0.28 (S)	0.28 (S)	0.63 (A)	0.63 (A)

*Isolate no.*, number of isolate; *FICI*, fractional inhibitory concentration index; *LNZ*, linezolid; *CYC*, cyclizine; *PIR*, piroxicam; *CCCP*, carbonyl cyanide m-chlorophenylhydrazine; *B*, vit B12; *NAC*, N-acetyl cysteine; *S*, synergy; *A*, additive

### Time–kill study

**Fig. 4:**
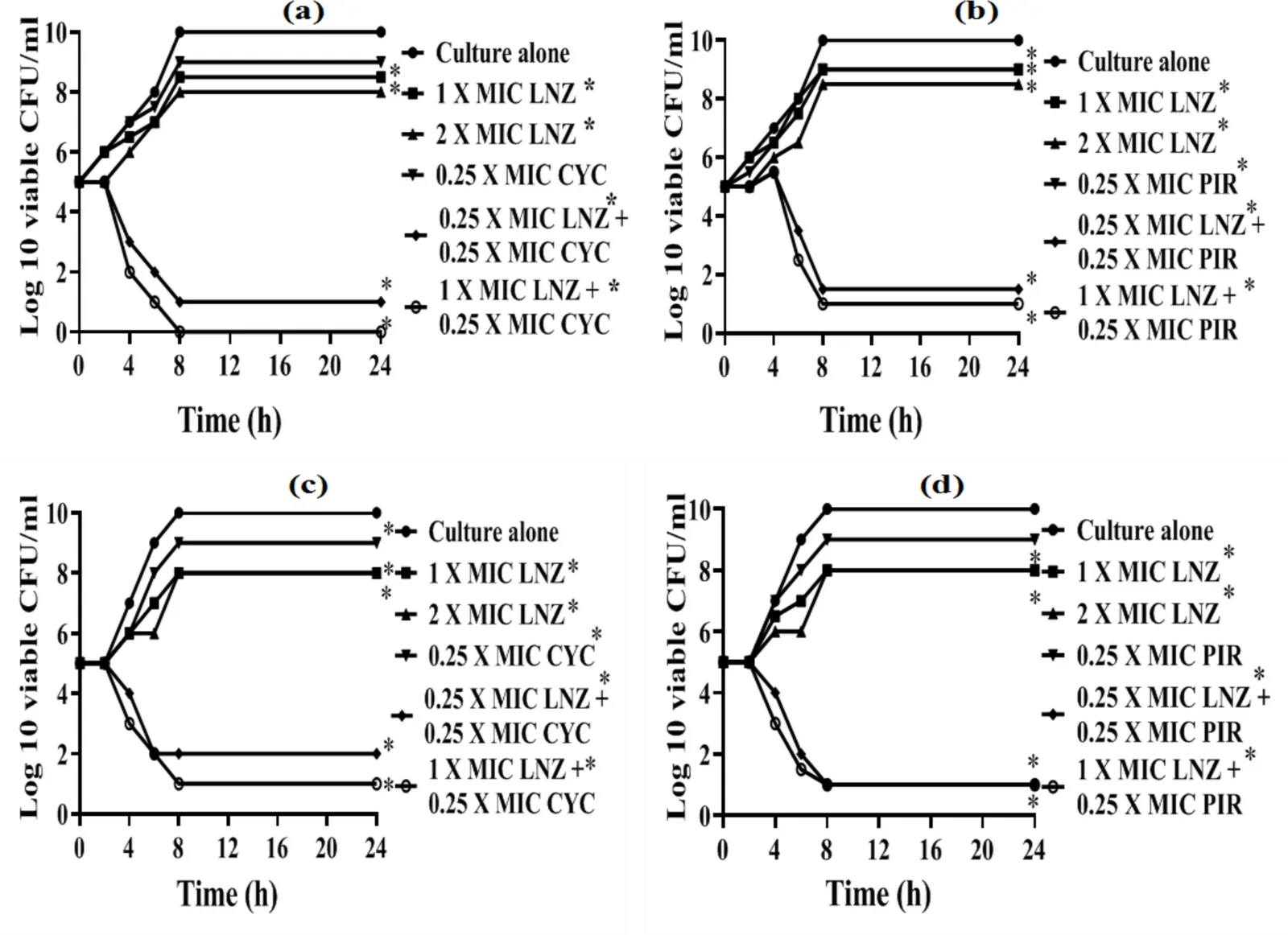
Time–kill curve. **a** Linezolid plus cyclizine combination against isolate no. 40. **b** Linezolid plus piroxicam combination against isolate no. 40. **c** Linezolid plus cyclizine combination against isolate no. 41. **d** Linezolid plus piroxicam combination against isolate no. 41. LNZ, linezolid; CYC, cyclizine; PIR, piroxicam; MICs, minimum inhibitory concentrations; CFU, colony-forming unit; h, hour; *probability value (*P* value) < 0.05, which is considered statistically significant

To confirm the synergistic effect of the two most promising compounds (cyclizine and piroxicam) with linezolid, time‒kill curves were generated with two LRSA isolates (no. 40 and 41). For the tested isolates treated with 1× MIC LNZ, 2× MIC LNZ, 0.25× MIC CYC, and 0.25× MIC PIR, the viable count significantly increased from 10^4^–10^5^ at time zero to 10^7^–10^10^ after 8–24 h. As expected, linezolid, cyclizine, and piroxicam alone had no bactericidal effect against the tested LRSA isolates (Fig. 4). The bacterial viable count significantly decreased from (10^4^–10^5^) at time zero to (10^2^–0) after 8–24 h when the isolates were subjected to the combined treatment (0.25× MIC LNZ + 0.25× MIC CYC), (1× MIC LNZ + 0.25× MIC CYC), (0.25× MIC LNZ + 0.25× MIC PIR), and (1× MIC LNZ + 0.25× MIC PIR). A reduction in bacterial count of at least 3 log10 CFU/ml was observed after 8–24 h of incubation with combinations of linezolid (1× MIC) with low concentrations of either cyclizine or piroxicam (0.25× MIC), resulting in a synergistic and bactericidal effect against the LRSA strains.

### In vivo evaluation of linezolid’s synergy with cyclizine and piroxicam

**Fig. 5:**
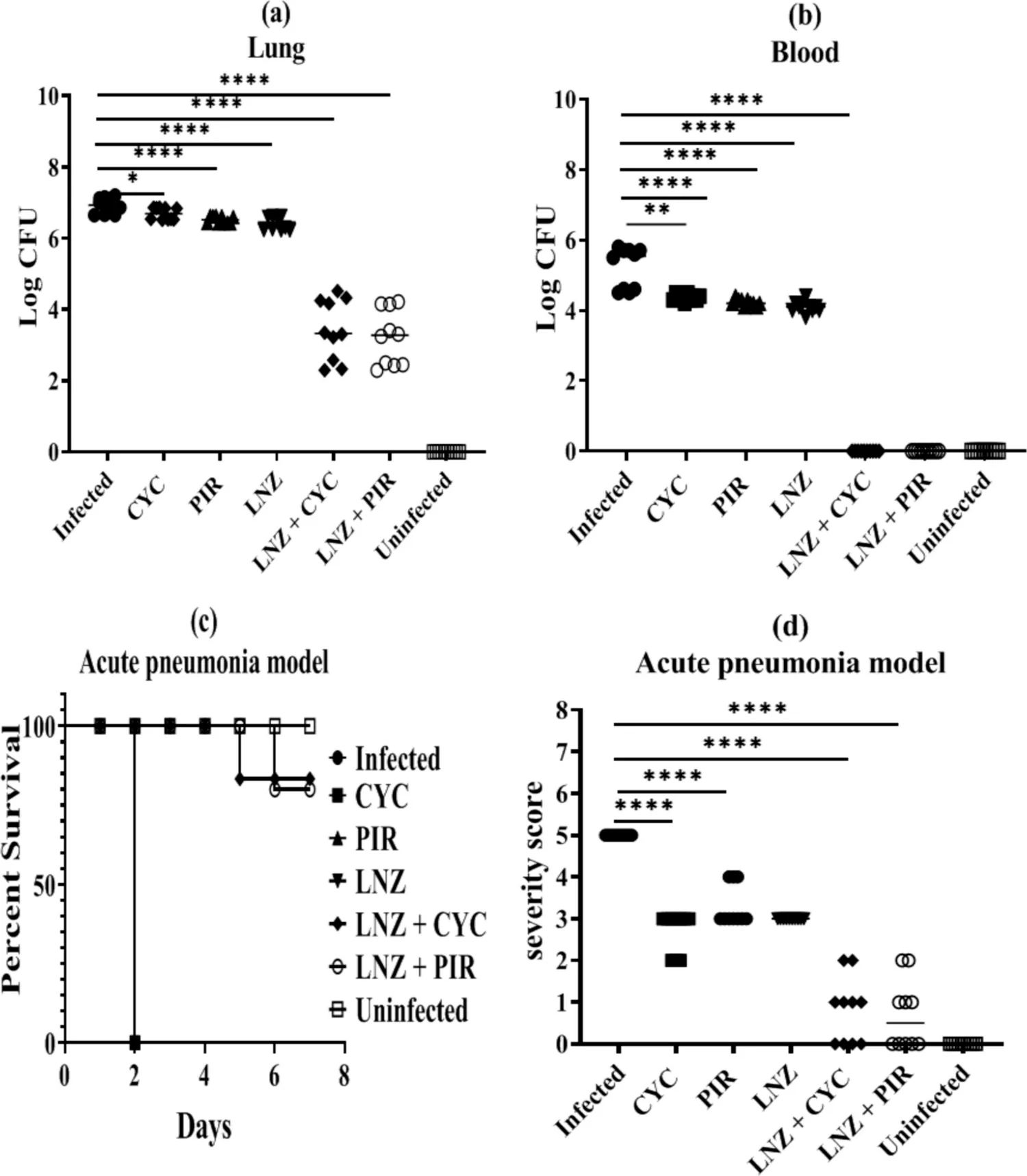
Linezolid combined with cyclizine and piroxicam reduced the severity of LRSA infection in the acute pneumonia model. **a **and **b** The mice groups that received combined treatments showed a significant reduction in viable bacteria in both lung tissues and blood compared to the groups that received single treatments or were left untreated. Data are presented as scatter plots. **c** Percentage survival against LRSA infection in the cyclizine, piroxicam, linezolid, linezolid combined with cyclizine, and linezolid combined with piroxicam groups. **d** Severity scores of lungs from control mice and mice with therapy 48 h after infection. Data are presented as scatter plots. ****P ≤ 0.0001. LNZ, linezolid; CYC, cyclizine; PIR, piroxicam

To further evaluate the synergy of linezolid with cyclizine and piroxicam, an in vivomouse model of acute pneumonia was performed. The mice were scored for disease progression and clinical signs throughout the study. Bacterial counts in the lung and blood of the mice were detected. A significant reduction in the bacterial count was observed in the lung tissues and blood samples collected from both combined treatment groups (LNZ + CYC and LNZ + PIR) compared with the single treatment and control groups. The most pronounced bacterial clearance was observed in the combined treatment groups, which presented significantly lower bacterial counts, with a (10^4^–10^6^)-fold decrease compared to the single treatment groups (Fig. 5 a and b).

Survival analysis revealed that all the mice from the infected, cyclizine, piroxicam, and linezolid groups died before 48 h after LRSA infection, with isolate no. 40 (LRSA40) with a high bacterial count in the lung tissues ranging from 4.2 × 10^7^ to 1.1 × 10^8^ CFU/g. Higher survival rates after infection were observed in the groups treated with linezolid combined with cyclizine (*P* = 0.0001) and linezolid combined with piroxicam (*P* = 0.0001) than in the single treatment groups. The significance of the therapeutic effect of linezolid combined with a low dose of cyclizine or piroxicam was measured using the log-rank test and ranged from 2.2 × 10^4^ to 8.3 × 10^3^ CFU/g (Fig. 5c). The average severity score (from zero to five) was significantly improved in the mice groups that received the combined treatment, ranging from 2 after 1 and 2 days post-infection to 1 after 5 days post-infection, reaching a score of zero after 7 days post-infection compared to those that received the single treatment, with scores ranging from 4 to 2 (Fig. 5d).

**Fig. 6:**
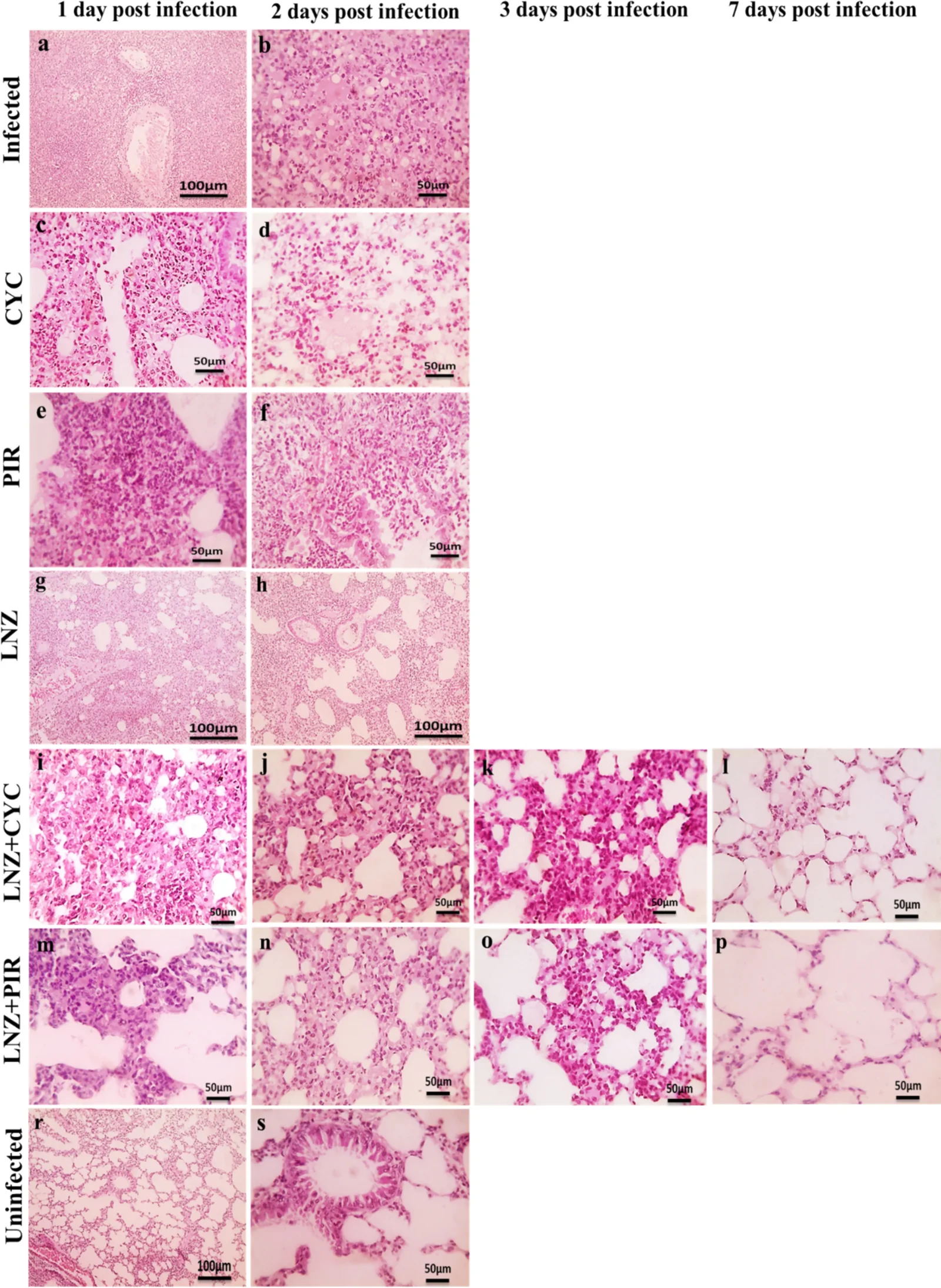
Linezolid’s synergy with cyclizine and piroxicam protects lung tissues of LRSA-challenged mice. Histological analysis of lung sections from mice treated with linezolid plus cyclizine (**i**, **j**, **k**, and **l**) and linezolid plus piroxicam (**m**, **n**, **o**, and **p**) showed a significant improvement in lung pathology compared to those treated with cyclizine (**c** and **d**), piroxicam (**e** and **f**), linezolid (**g** and **h**), and untreated (**a** and **b**). Lung sections from normal, non-infected mice were used as control (**q**)

Histopathological examination of lung sections from the control mouse group and the cyclizine, piroxicam, and linezolid treatment groups 2 days after infection revealed extensive tissue damage characterized by vascular congestion; severe pneumonia with decreased air space; and multifocal accumulation of inflammatory exudate infiltrated with polymorphonuclear cells and perivascular lymphocytic cell aggregation (Fig. 6 b, d, f, and h). In contrast, significant improvements in lung pathology during the early post-infection period were observed in lung sections from mice treated with linezolid plus cyclizine and linezolid plus piroxicam, as evidenced by decreased pneumonia, reduced inflammatory exudate, and fewer polymorphonuclear cells compared to the control group (Fig. 6 j and n). Furthermore, on day 7 post infection, no histopathological abnormalities were detected in the lung tissues from the combined treatment groups, as they presented a normal air space and normal alveolar wall (Fig. 6 l and p). These findings correlate with lung sections showing no structural damage, inflammation, or congestion, similar to those of the uninfected group, which had normal lung cells (Fig. 6q).

## Discussion

Linezolid is considered a last resort for treating severe gram-positive infections (Yang et al. 2025). Major linezolid resistance mechanisms against *S. aureus* incorporate mutations in domain V of 23S rRNA, chromosomal mutations in ribosomal protein genes *(rplC*, *rplD*, and *rplV*), acquisition of the *cfr* genes encoding a methylase, and the presence of resistance genes (*optrA* and *poxtA*) and the LmrS multidrug efflux pump (Valderrama et al. 2020). In recent years, the increasing prevalence of linezolid-resistant *S. aureus* (LRSA) has been due to the widespread use of linezolid (Zhou et al. 2025). This warrants the development of novel treatment strategies to effectively inhibit these resistant strains, such as combination therapies. While various in vitro studies have explored combination therapies, including linezolid, only a limited number of research studies have examined the in vivo efficacy of combinations of linezolid with other drugs (Jacqueline et al. 2005).

Among the *S. aureus* isolates, a high prevalence of MRSA isolates (97.73%) was detected; these isolates were cefoxitin-resistant and carried the *mecA* gene. Similar findings were reported in Egypt (Mahsaly, El Mashad et al. 2019). This is consistent with the fact that MRSA resistance is attributed mainly to the presence of the *mecA* gene, which encodes altered penicillin-binding protein 2a (PBP2a) (Zhou et al. 2025).

Vancomycin, a glycopeptide antibiotic commonly used to treat resistant gram-positive infections (Bayer et al. 2013). In our study, 38.64% of the isolates were identified as VRSA using the broth microdilution method, consistent with previous studies in Egypt showing a similar prevalence of VRSA (34%) (Abdelraheem et al. 2021). Other studies in Egypt have reported high VRSA prevalence rates of 23.62% (Ibrahiem, Rizket al. 2022) VRSA prevalence is also high in Asia and Africa, with Nigeria and Saudi Arabia reporting rates of up to 29% and 18%, respectively (Alzolibani, Al Robaee et al. 2012, Olufunmiso et al. 2017). India and Pakistan have reported significant numbers of VRSA strains, with rates as high as 16% to 33% in specific tertiary care settings Mendem (Gangadhara et al. 2016; Wu, Sabokroo et al. 2021).

Our analysis also showed that 22.73% of the *S. aureus* isolates were LRSA, identified through susceptibility testing to linezolid using the disc diffusion method and PCR detection of the *optrA*, *cfr*, and *cfr(B*) genes. Similar rates of LRSA have been reported in Egypt (Aamir et al. 2015). Various studies over the past decade have reported an increase in LRSA isolates, mainly due to the inappropriate use of linezolid (Ashour and el-Sharif 2007; ElSayed et al. 2018). Additionally, a previous study in Egypt indicated that a higher prevalence of LRSA isolates (29.31%) was found in the 116 cases examined (Abdelraheem et al. 2021). In contrast, the current findings demonstrated a higher prevalence of LRSA than the global rates documented in the LEADER or ZAAPS studies (Jones, Ross et al. 2008, Farrell, Mendes et al. 2009, Jones, Kohno et al. 2009). The LRSA prevalence among *S. aureus* species in the ZAAPS/LEADER studies was only 0.10%–0.02% (Jones, Kohno et al.^2009^, Ross, Farrell et al. 2011). This could be attributed to the linezolid’s availability in the Egyptian market, its use as an empirical therapy, and the lack of guidelines for controlling its use. This misuse has contributed to the dissemination of resistance in *Staphylococcus* (Rafique, Hussain et al. 2022).

The *optrA* gene was detected in 9.09% of the 44 *S. aureus* isolates, whereas the *cfr* and *cfr(B)* genes were not detected. The *optrA* gene is an ATP-binding cassette F (ABC-F) protein that binds to the 50S ribosomal subunit and disrupts its structure, preventing linezolid from binding to the ribosome. This directly affects the function of the ribosome by altering the PTC, where antibiotics typically bind to its functional site (Wang et al. 2016; Wilson 2016, Ruiz-Ripa, Feßler et al. 2020). Previous studies reported the presence of the *optrA* gene and the absence of the *cfr* gene in linezolid-resistant isolates, which aligns with the findings of our study (Ma et al. 2021; Elaskary and Zaher 2022). Other studies detected the *optrA* and *cfr* genes in 80% and 78% of linezolid-resistant isolates, respectively (Azhar et al. 2017; Said and Abdelmegeed 2019). *OptrA* and *cfr* are significant resistance genes in gram-positive bacteria, particularly *Staphylococcus* and *Enterococcus*, contributing to multidrug resistance and complicating the treatment of severe infections (Antonelli, D’Andrea et al. 2018). Both can be found together on the same mobile elements (plasmids) in both *Enterococcus* and *Staphylococcus* species, increasing resistance concerns with the same mechanism (Suo, Yu et al. 2025).

The *cfr* gene and its variant *cfr(B*) gene encode an rRNA methyltransferase that showed resistance against a wide range of antimicrobial agents targeting the peptidyl transferase center (PTC). Resistance is achieved by C8-methylation of A2503, the highly conserved adenosine residue in the 23S ribosomal RNA (Yang et al. 2025). Contrary to our results, previous studies have reported a high expression rate of the *cfr* gene in 85.30% (29/34) of LRSA isolates (Abdelraheem et al. 2021). In addition, the *cfr*-mediated resistance gene was found in all the LRSA isolates (Morales et al. 2010). In line with our results, the *cfr* gene was not detected in any of the isolates, while three out of 159 isolates were found to have the *cfr(B)* gene (1.8%) (AbdAlhafiz et al. 2023).

Our data revealed that 23% of the 44 *S. aureus* isolates were obtained from wound infections. This was in accordance with other studies, which reported that wounds were the most prevalent source for MRSA and VRSA infections (Razeghi et al. 2019; Almuhayawi et al. 2023).

The treatment of infections caused by *S. aureus* has become increasingly challenging because of its resistance to multiple antimicrobial drugs (Garoy et al. 2019). Previous in vitro studies have investigated the effects of linezolid with various antimicrobial agents, such as daptomycin, gentamicin, erythromycin, tetracycline, imipenem, and plazomicin, on MRSA isolates (Sweeney and Zurenko 2003; Ribes, Pachón-Ibáñez et al. (Ribes 2010a,b; Lee et al. 2019; Valderrama et al. 2020).

The in vitro evaluation of linezolid with different compounds was initially performed using different methods. We first assessed the synergistic effects of sub-MICs of these compounds in combination with linezolid using the broth microdilution method. The checkerboard method was then used to determine the potential synergy between drug combinations and to identify the optimal concentrations of linezolid in combination with synergistic compounds for further evaluation through time–kill curve experiments (Valderrama et al. 2020). Finally, a time–kill study was utilized for evaluating the in vitro efficacy of the drug combinations (Valderrama et al. 2020). Our data revealed that the best combinations were linezolid plus cyclizine and linezolid plus piroxicam.

Using the broth microdilution method, the minimum inhibitory concentration of linezolid significantly decreased by 64–128 times when combined with the sub-MIC of cyclizine against the four LRSA isolates (Supplementary Table 4 and Fig. 4). The linezolid/cyclizine combination exhibited a synergistic effect against the four tested isolates using the checkerboard method, which was confirmed by the combination’s synergistic and bactericidal activity, as indicated by time‒kill curves (Table 1 and Fig. 5c). In parallel, the addition of a sub-MIC of piroxicam, a NSAID, decreased the MIC of linezolid against all the isolates by 64–128 times. The synergistic effect was confirmed by a checkerboard with FICIs ranging from 0.28 to 0.5 and time–kill methods. Synergistic effects were observed, potentially due to piroxicam’s inhibition of *S. aureus* adherence (Roux et al. 2013; Bhardwaj et al. 2020)*.*

In accordance with our results, several studies have shown that linezolid combined with imipenem (FICI = 0.187 to 0.59) has a synergistic effect against *S. aureus*, including MRSA (Valderrama et al. 2020). Another study demonstrated synergy when subinhibitory levels of ertapenem with linezolid were used at concentrations 2 to 8 times greater than the MIC for both strains. In the case of MRSA, a combination of ertapenem at 1/128× MIC and linezolid at 4× MIC strongly decreased the number of bacteria compared with the most effective single antibiotic (Jacqueline et al. 2006).

However, combination therapy, which involves the use of one antibiotic along with another non-antibiotic drug, is often more effective in treating bacterial infections than the use of combinations of two antibiotics. This strategy can help overcome antibiotic resistance, minimize side effects, and improve treatment effectiveness (Sullivan et al. 2020; Wang et al. 2022).

Since animal models are necessary for validating the combination activity identified in vitro (Jacqueline et al. 2005), this study employed an in vivo acute pneumonia mouse model to assess the effectiveness of combining linezolid with cyclizine and piroxicam in treating LR-MRSA infections compared with the use of each drug individually.

Pneumonia mouse models were previously used in a study of linezolid/rifampicin combination (Zhou,Xiong et al. 2018), linezolid/vancomycin or imipenem combinations (Ribes, Pachón-Ibáñez et al. 2010a, b, Hamed, Arafa et al. 2022), and linezolid/ertapenem combination (Jacqueline et al. 2006) in treating pneumonia caused by MRSA.

When a high dose of this strain was administered intranasally to mice, it led to severe lung infection and bacteremia within 24 h. Initially, single treatments with two doses of either intranasal linezolid, intraperitoneal cyclizine, or intranasal piroxicam did not significantly affect *S. aureus* infection compared with the untreated control group. However, when two doses of intranasal linezolid were combined with low doses of intraperitoneal cyclizine or intranasal piroxicam, there was a significant increase in survival rates and a significant reduction in bacterial colonization of approximately 3–4 log10 CFU/g in the lungs of infected mice compared with the infected positive control counts. Furthermore, the combination of intranasal linezolid with intraperitoneal cyclizine or intranasal piroxicam led to better histopathological outcomes and a significant improvement in the clinical score. Our findings suggest that the combination of intranasal linezolid with either intraperitoneal cyclizine or intranasal piroxicam effectively eradicated bacteria from the blood, promoted the recovery of *S. aureus* infection, and extended the survival time of infected mice.

The synergism of the linezolid/cyclizine combination may contribute to the inhibitory activity of cyclizine against linezolid resistance. The combined effect of linezolid and cyclizine may be attributed to the antimicrobial properties of cyclizine, an antihistaminic and antiemetic drug. Cyclizine has exhibited in vitro antimycobacterial activity against *Mycobacterium* *abscessus* isolates obtained from cystic fibrosis patients (Kirkwood et al. 2018). Alternatively, the synergistic activity of the linezolid/piroxicam combination can be attributed to its ability to inhibit the adherence of *S. aureus*, reinforcing its activity with linezolid. Piroxicam, similar to other NSAIDs, has pain-relieving, fever-reducing, and anti-inflammatory effects. Piroxicam blocks tissue cyclooxygenases (Cox-1 and −2) and reduces the production of pro-inflammatory prostaglandins which lead to pain and inflammation (Roux et al. 2013; Bhardwaj et al. 2020). The inflammatory process and protective reactions of the body may be influenced by the production of the pro-inflammatory prostaglandin PGE2, as the bacterial growth is stimulated by the synthesis of the prostaglandin PGE2 by fungi, and NSAIDs inhibit this effect (Abd El-Baky 2015; Rumynska et al. 2021).

A previous in vivo study revealed that the use of linezolid in combination with vancomycin or imipenem had greater efficacy in decreasing bacterial colonization and mortality rates due to pneumonia in mice infected with heterogeneous glycopeptide-intermediate *S. aureus* (hGISA) compared to using these antibiotics alone or in combinations (Duan et al. 2021). Another in vivo study reported that the use of monoclonal antibodies (mAbs) with low doses of linezolid improved lung scores and increased survival rates in an acute pneumonia mouse model. This combination decreased the bacterial count in the lungs of the mice and maintained normal physiological architecture (Ribes, Pachón-Ibáñez et al. 2010a, b). In addition, ertapenem with linezolid combination therapy demonstrated in vivosynergistic and bactericidal effects against MRSA strains, in contrast to the lack of activity when either drug was used alone (Jacqueline et al. 2006).

The therapeutic landscape for MDR-MRSA is now evolving from reliance on single-agent glycopeptides toward tailored combinations (Mahjabeen, Saha et al. 2022, Kumar et al. 2025). Combination therapy, using one antibiotic with a non-antibiotic drug, is often more effective against bacterial infections than using two antibiotics (Chan, Yee et al. 2017, Wang et al., 2022). Our study confirmed the synergistic effects of combining linezolid with two non-antimicrobial drugs: cyclizine or piroxicam against LRSA infections. These drugs are readily available, safe, and cost-effective, making them promising candidates for inclusion in hospital antibiotic protocols to enhance treatment outcomes, combat resistance, minimize side effects, and improve overall effectiveness after conducting adequate studies for clinical implementation.

Previous research has focused mainly on the use of dual antibiotic combinations for the treatment of severe LRSA infections. The main strength of this study is the innovative synergistic combinations of linezolid with cyclizine or piroxicam against LRSA infection with in vitroand in vivo activity. Additionally, all the drugs used are safe and approved by the Food and Drug Administration. The limitation of this study is that more research is required to investigate the synergistic mechanisms between these drugs and linezolid. Also, further preclinical studies are required to confirm the safety and efficacy of these combinations before moving forward with clinical trials.

## Conclusion

Our data revealed that using linezolid in combination with sub-inhibitory concentrations of cyclizine and piroxicam showed bactericidal effects against LR-MRSA strains after two doses of this therapy. Clinically achievable concentrations of both the antibiotic and the synergistic compounds were used. Linezolid in combination with cyclizine or piroxicam seems to have promising results for the treatment of severe LR-MRSA infections and warrants more research. For more interpretation and understanding of the synergistic mechanisms between the two combinations, this study requires further investigation, and the clinical implications of our results need to be assessed.

## Supplementary Information

Below is the link to the electronic supplementary material.

ESM 1 (PDF 1.00 MB)

## Data Availability

All the data developed or analyzed during this study, which included in the manuscript and the supplementary information files can be obtained from the corresponding author upon reasonable request.

## Abbreviations

MRSA: Methicillin-resistant *Staphylococcus aureus*
LRSA: Linezolid-resistant *Staphylococcus aureus*
VRSA: Vancomycin-resistant *Staphylococcus aureus*
*S. aureus*: *Staphylococcus* *aureus*
TSS: Toxic shock syndrome
FDA: Food and Drug Administration
UTIs: Urinary tract infections
PTC: Peptidyl transferase center
LR-MRSA: Linezolid-resistant MRSA
WHO: World Health Organization
MIC: Minimum inhibitory concentration
PCR: Polymerase chain reaction
TTC: Tetrazolium chloride
FIC: Fractional inhibitory concentration
FICI: FIC index
MH broth: Mueller–Hinton Broth
CFU: Colony-forming unit
CCCP: Carbonyl cyanide m-chlorophenylhydrazine
*LmrS*: Lincomycin resistance protein of *Staphylococcus aureus*
ZAAPS: Zyvox annual appraisal of potency and spectrum
LEADER: Linezolid experience and accurate determination of resistance
ABC-F: ATP-binding cassette F
Sub-MIC: Subinhibitory concentration
mAb: Monoclonal antibodies
