# Supplementary material for: In vitro and in vivo synergistic effects of cyclizine and piroxicam in combination with linezolid against methicillin-resistant Staphylococcus aureus
Source: Appl Microbiol Biotechnol. 2026 Mar 19;110(1):109. doi: 10.1007/s00253-026-13738-9 (PMC13004739; doi:10.1007/s00253-026-13738-9)
Supplement: Supplementary file 1 — (PDF 1.00 MB) [file 253_2026_13738_MOESM1_ESM.pdf]

In Vitro and In Vivo Synergistic Effects of Cyclizine and Piroxicam in Combination with Linezolid against Methicillin-Resistant *Staphylococcus aureus*

Mai , Abeer M. Abd El-Aziz, Mona I. Shaaban

Department of Microbiology and Immunology, Faculty of Pharmacy, Mansoura University, Mansoura 35516, Egypt

***Corresponding author:** Mona I. Shaaban

mona_ibrahem@mans.edu.eg

Supplementary table 1. Antimicrobial susceptibility of the tested *S. aureus* clinical isolates to different classes of antimicrobial agents.

Isolate No.	Clinical source	β-lactams				Aminoglycosides	Tetracyclins	Fluoroquinolones	Macrolides	Lincosamides	Oxazolidones
		AMC	FOX	CTX	IPM	CN	DO	LEV	E	DA	LNZ
1	Diabetic foot	R	R	R	S	S	I	R	S	S	S
2	Diabetic foot	R	R	R	S	S	S	S	I	S	S
3	Eye swab	R	R	R	S	S	R	S	R	R	S
4	Eye swab	S	R	R	S	S	R	S	R	S	S
5	Eye swab	R	R	R	S	R	R	S	R	I	S
6	Eye swab	R	R	R	S	S	I	S	R	R	S
7	Eye swab	S	R	R	S	I	R	S	R	S	S
8	Eye swab	R	R	R	S	S	R	S	R	R	S
9	Eye swab	R	R	R	S	R	S	R	I	S	S
10	Nasal discharge	R	R	R	S	R	R	S	I	S	S
11	Nasal discharge	R	R	R	S	S	R	S	I	S	S
12	Nasal discharge	R	R	R	S	R	R	S	I	S	S
13	Nasal discharge	R	R	R	S	R	R	S	S	I	S
14	Sputum	R	S	R	S	S	S	S	S	R	S
15	Sputum	R	R	R	R	R	R	R	R	S	S
16	Sputum	R	R	R	S	S	R	S	I	S	S
17	Sputum	R	R	R	S	S	R	S	R	S	S
18	Sputum	R	R	R	S	S	S	S	S	I	S
19	Sputum	R	R	R	S	S	S	R	S	S	S
20	Sputum	R	R	R	S	S	S	S	R	S	S
21	Sputum	R	R	R	S	S	R	S	S	S	S
22	Sputum	R	R	I	R	R	S	S	I	R	R

Isolate No.	Clinical source	β-lactams				Aminoglycosides	Tetracyclins	Fluoroquinolones	Macrolides	Lincosamides	Oxazolidoness
		AMC	FOX	CTX	IPM	CN	DO	LEV	E	DA	LNZ
23	Blood	R	R	R	S	I	S	R	R	S	S
24	Blood	R	R	R	S	S	S	S	R	S	S
25	Blood	R	R	R	S	S	R	R	R	S	S
26	Blood	R	R	R	S	S	S	S	R	S	S
27	Blood	R	R	R	R	R	I	R	R	R	S
28	Blood	R	R	R	S	R	I	S	S	S	S
29	Blood	R	R	R	S	S	I	S	S	S	S
30	Blood	R	R	R	R	R	R	R	R	R	R
31	Urine	R	R	R	S	S	R	R	R	S	S
32	Urine	R	R	R	S	S	S	S	I	S	S
33	Urine	R	R	I	S	S	S	S	S	S	S
34	Urine	R	R	R	S	S	S	S	R	S	S
35	Wound	R	R	R	R	R	R	S	R	R	R
36	Wound	R	R	R	R	R	R	R	R	R	R
37	Wound	R	R	R	R	R	S	S	R	R	R
38	Wound	R	R	R	S	S	R	R	R	R	S
39	Wound	R	R	R	R	I	R	R	R	R	R
40	Wound	R	R	R	R	R	R	R	R	R	R
41	Wound	R	R	R	R	R	R	R	R	R	R
42	Wound	R	R	R	R	R	I	R	S	R	S
43	Wound	R	R	R	S	S	R	R	I	R	R
44	Wound	R	R	R	S	S	R	R	R	R	R

Isolate No.: the number of isolate, **AMC:** amoxicillin-clavulanic acid, **FOX:** cefoxitin, **CTX:** cefotaxime, **IPM:** imipenem, **CN:** gentamicin, **DO**: doxycycline, **LEV:** levofloxacin, **E:** erythromycin, **DA:** clindamycin, **LNZ:** linezolid, **S:** susceptible, **I:** intermediate, **R:** resistant.

Supplementary table 2. Classification of *S. aureus* isolates according to susceptibility to vancomycin

Isolate No.	MIC (µg/ml)	Type	Isolate No.	MIC (µg/ml)	Type
1	> 64	VRSA	23	< 2	VSSA
2	> 64	VRSA	24	< 2	VSSA
3	< 2	VSSA	25	> 64	VRSA
4	4	VISA	26	2	VSSA
5	4	VISA	27	< 2	VSSA
6	< 2	VSSA	28	> 64	VRSA
7	8	VISA	29	> 64	VRSA
8	4	VISA	30	> 64	VRSA
9	8	VISA	31	4	VISA
10	> 64	VRSA	32	4	VISA
11	> 64	VRSA	33	8	VISA
12	< 2	VSSA	34	< 2	VSSA
13	4	VISA	35	> 64	VRSA
14	< 2	VSSA	36	> 64	VRSA
15	< 2	VSSA	37	> 64	VRSA
16	8	VISA	38	8	VISA
17	8	VISA	39	< 2	VSSA
18	> 64	VRSA	40	> 64	VRSA
19	8	VISA	41	> 64	VRSA
20	8	VISA	42	4	VISA
21	8	VISA	43	> 64	VRSA
22	> 64	VRSA	44	> 64	VRSA

Isolate No.: number of isolate, **MIC**: minimum inhibitory concentration **VSSA**: vancomycin-sensitive *S. aureus* **VISA**: vancomycin-intermediate *S. aureus* **VRSA**: vancomycin-resistant *S. aureus.*

Supplementary table 3. Prevalence of genes conferring resistance to methicillin and linezolid in *S. aureus* isolates

Isolate No.	*mecA*	*optrA*	*cfr*	*CfrB*	Isolate No.	*mecA*	*optrA*	*cfr*	*CfrB*
1	+	-	-	-	23	+	-	-	-
2	+	-	-	-	24	+	-	-	-
3	+	-	-	-	25	+	-	-	-
4	+	-	-	-	26	+	-	-	-
5	+	-	-	-	27	+	-	-	-
6	+	-	-	-	28	+	-	-	-
7	+	-	-	-	29	+	-	-	-
8	+	-	-	-	30	+	-	-	-
9	+	-	-	-	31	+	-	-	-
10	+	-	-	-	32	+	-	-	-
11	+	-	-	-	33	+	-	-	-
12	+	-	-	-	34	+	-	-	-
13	+	-	-	-	35	+	+	-	-
14	-	-	-	-	36	+	-	-	-
15	+	-	-	-	37	+	-	-	-
16	+	-	-	-	38	+	-	-	-
17	+	-	-	-	39	+	-	-	-
18	+	-	-	-	40	+	+	-	-
19	+	-	-	-	41	+	+	-	-
20	+	-	-	-	42	+	-	-	-
21	+	-	-	-	43	+	-	-	-
22	+	+	-	-	44	+	-	-	-

Isolate No.: number of isolate.

Supplementary table 4. The effect of WHO-approved compounds on linezolid resistance against *S. aureus* isolates

Isolate No.	MICs of LNZ (µg/ml)	Potential inhibitor	MICs of inhibitor (µg/ml)	Sub-MIC of inhibitor (µg/ml)	MICs of LNZ with inhibitor (µg/ml)	Fold change	*P*-value
22	512	Cyclizine (CYC)	4693.75	2346.875	4	**128 fold (-)**	0.0013**
35	256		2346.875	1173.438	2	**128 fold (-)**	0.0013**
40	512		4693.75	2346.875	8	**64 fold (-)**	0.0025**
41	256		4693.75	2346.875	4	**64 fold (-)**	0.0025**
22	512	Piroxicam (PIR)	5000	2500	4	**128 fold (-)**	0.0013**
35	256		5000	2500	4	**64 fold (-)**	0.0025**
40	512		2500	1250	4	**128 fold (-)**	0.0013**
41	256		5000	2500	4	**64 fold (-)**	0.0025**
22	512	VitB12 (B)	1000	500	128	**4 fold (-)**	0.0489*
35	256		500	250	64	**4 fold (-)**	0.0489*
40	512		500	250	64	**8 fold (-)**	0.0219*
41	256		500	250	32	**8 fold (-)**	0.0219*
22	512	(CCCP)	100	50	64	**8 fold (-)**	0.0219*
35	256		50	25	32	**8 fold (-)**	0.0219*
40	512		100	50	32	**16 fold (-)**	0.0104*
41	256		100	50	16	**16 fold (-)**	0.0104*
22	512	N-acetyl cysteine (NAC)	50000	25000	128	**4 fold (-)**	0.0489*
35	256		50000	25000	64	**4 fold (-)**	0.0489*
40	512		50000	25000	32	**16 fold (-)**	0.0104*
41	256		50000	25000	32	**8 fold (-)**	0.0219*
22	512	Ondansetron (OND)	4000	2000	128	**4 fold (-)**	0.0489*
35	256		4000	2000	32	**8 fold (-)**	0.0219*
40	512		4000	2000	256	**2 fold (-)**	0.1257
41	256		4000	2000	64	**4 fold (-)**	0.0489*
22	512	Hyoscine-n-butyl bromide (HBB)	20000	10000	256	**2 fold (-)**	0.1257
35	256		40000	20000	64	**4 fold (-)**	0.0489*
40	512		40000	20000	256	**2 fold (-)**	0.1257
41	256		20000	10000	64	**4 fold (-)**	0.0489*
22	512	Dexamethasone (DEX)	4000	2000	256	**2 fold (-)**	0.1257
35	256		4000	2000	128	**2 fold (-)**	0.1257
40	512		2000	1000	256	**2 fold (-)**	0.1257
41	256		2000	1000	128	**2 fold (-)**	0.1257

22	512	L-carnitine (LC)	200000	100000	512	**-**	0.5000
35	256		200000	100000	256	**-**	0.5000
40	512		100000	50000	256	**2 fold (-)**	0.1257
41	256		400000	200000	128	**2 fold (-)**	0.1257

Isolate No.: number of isolate, **MICs:** minimum inhibitory concentrations, **LNZ:** linezolid, **CCCP:** carbonyl cyanide m-chlorophenylhydrazine, **(+);** increase, **conc.:** concentration, **(-):** decrease, ***P*-value:** probability-value, *: probability-value is <0.05 which is considered statistically significant.

Supplementary table 5. Primers used for detection of some resistance genes of *S. aureus* used in this study.

Type	Name	Nucleotide sequence (5` to 3`)	AT (˚C)	Amplicon size (bp)	Reference
**Methicillin resistance genes**	***mecA*** Fw<br>***mecA*** Rv	TGCTATCCACCCTCAAACAGG<br>AACGTTGTAACCACCCCAAGA	50	286	**(Kondo, Ito et al. 2007)**
**Linezolid resistance genes**	***Cfr*** Fw<br>***Cfr*** Rv	TGAAGTATAAAGCAGGTTGGG AGTCA<br>ACCATATAATTGACCACAAGC AGC G	56	747	**(Ibrahiem , Rizk et al. 2023)**
	cfrB Fw<br>*cfrB* Rv	CTCCTTTTAATGAACAGCGAA GTGAGT<br>GACCGCAAGCAGCGTCTATAT CA	58	375	**(Ibrahiem , Rizk et al. 2023)**
	optrA Fw<br>***optrA*** Rv	GGTGGATGAAGTCCGTACGGT A<br>CAAGGTGGTTAGTAGGTTCAT CAAG	57	320	**(Ibrahiem , Rizk et al. 2023)**

AT: annealing temperature, **bp:** base pair, **Fw:** forward, **Rv:** reverse.

Supplementary table 6. Mice groups, drugs, doses received, and their route of administration.

**Group name**	**Drug, dose, and route of administration**
Infected Group	The infection only without the treatment (positive control)
CYC Group	Intraperitoneal cyclizine (1.41 mg/mouse)
PIR Group	Intranasal piroxicam (0.04 mg/mouse)
LNZ Group	Intranasal linezolid (0.1 mg/mouse)
LNZ+CYC Group	Intranasal linezolid (0.1 mg/mouse) plus intraperitoneal cyclizine (1.4 mg/mouse)
LNZ+ PIR Group	Intranasal linezolid (0.1 mg/mouse) plus intranasal piroxicam (0.04 mg/mouse)
Uninfected Group	Saline only without the treatment **(negative control)**

CYC: cyclizine, **PIR:** piroxicam, **LNZ:** linezolid.

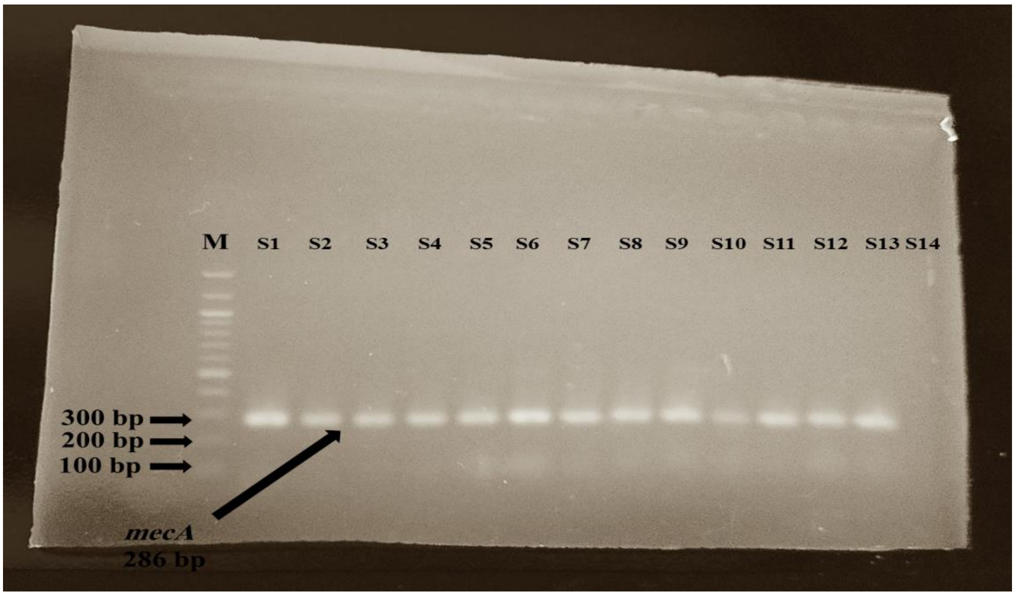

Supplementary Fig. 1.a: Agarose gel electrophoresis representing *mecA* gene among some of the tested isolates (1-14) with amplicon size 286 bp.

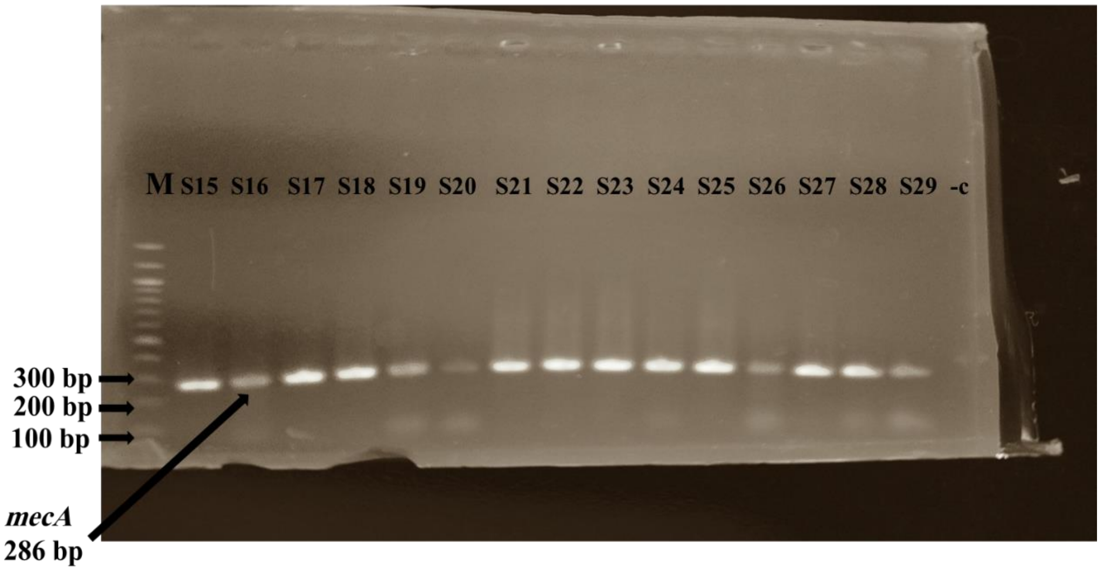

Supplementary Fig. 1.b: Agarose gel electrophoresis representing *mecA* gene among some of the tested isolates (15-29) with amplicon size 286 bp.

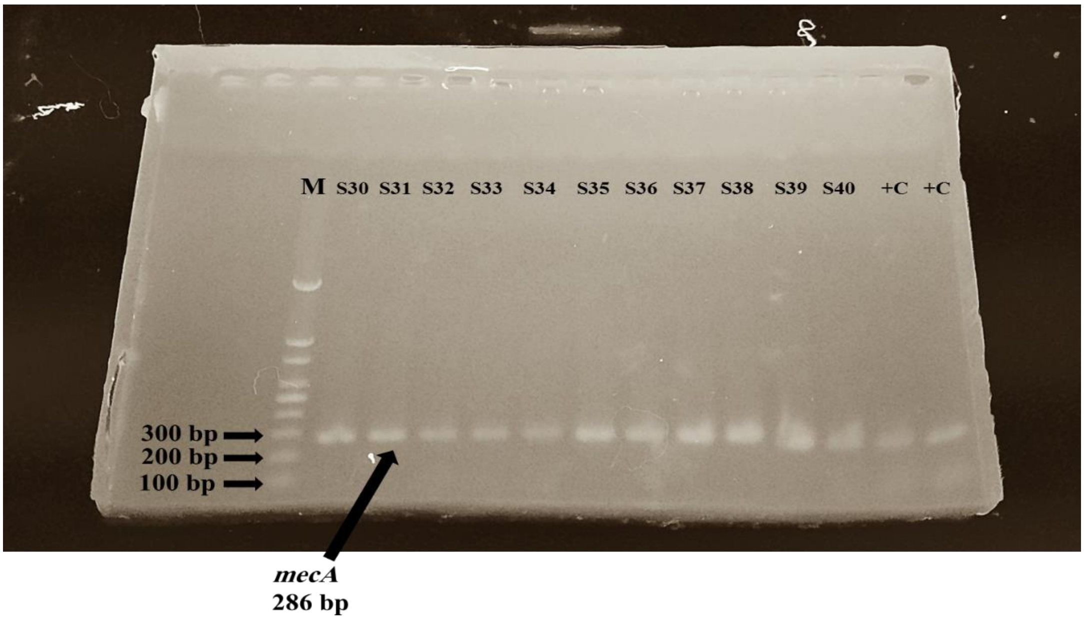

Supplementary Fig. 1.c: Agarose gel electrophoresis representing *mecA* gene among some of the tested isolates (30-40) with amplicon size 286 bp.

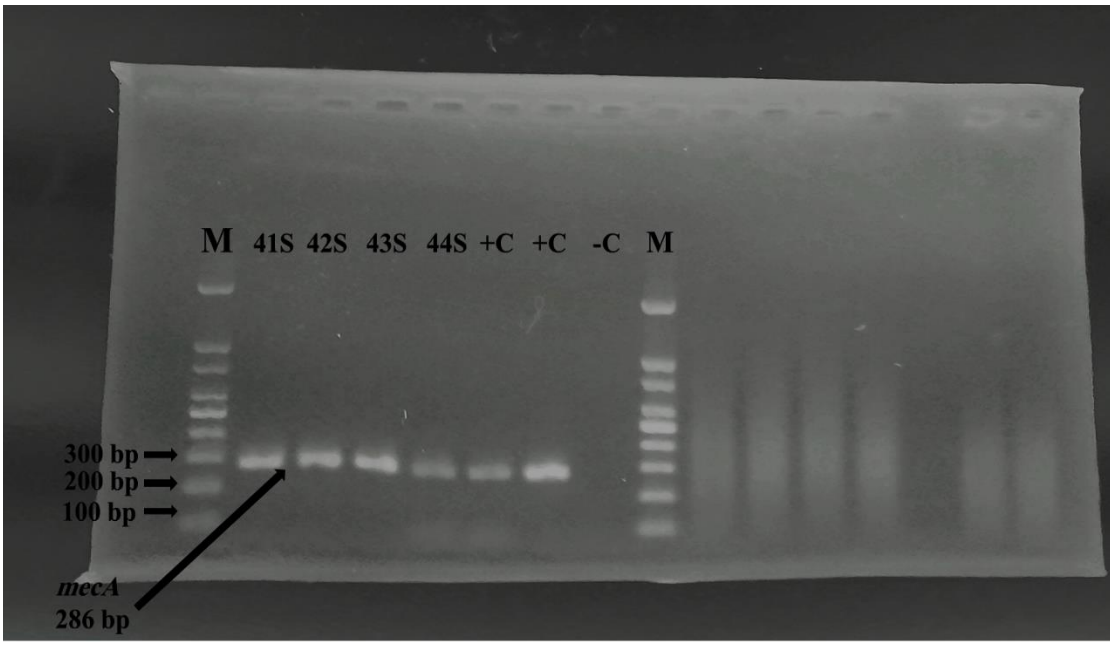

Supplementary Fig. 1.d: Agarose gel electrophoresis representing *mecA* gene among some of the tested isolates (41-44) with amplicon size 286 bp.

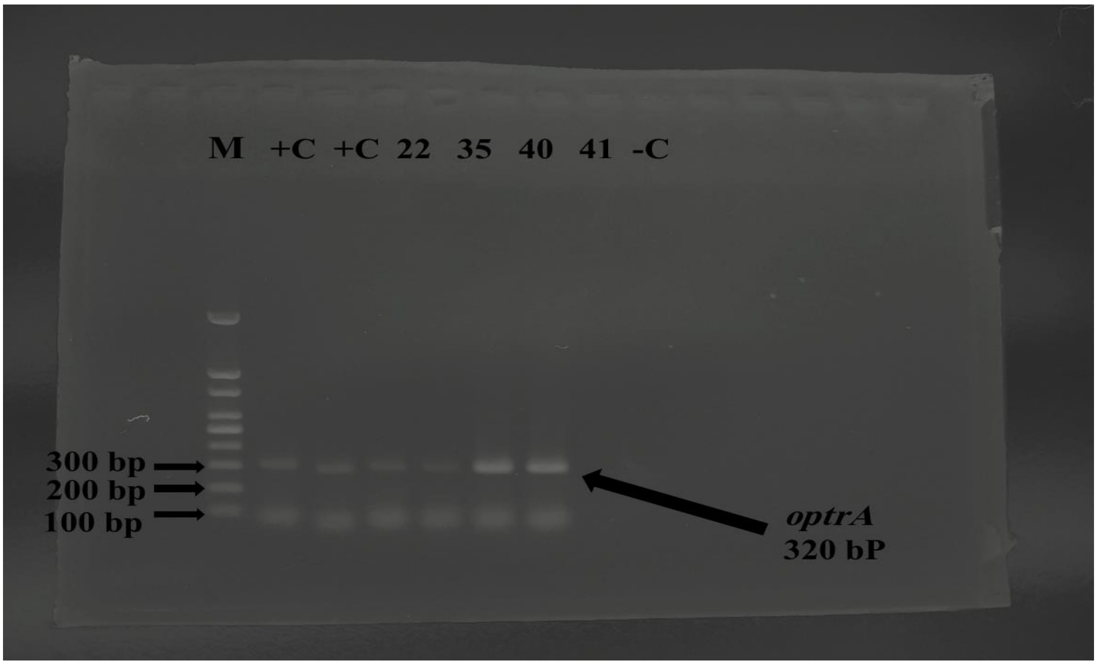

Supplementary Fig. 2: Agarose gel electrophoresis representing *optrA* gene among some of the tested isolated 22, 35, 40 and 41 with amplicon size 320 bp.

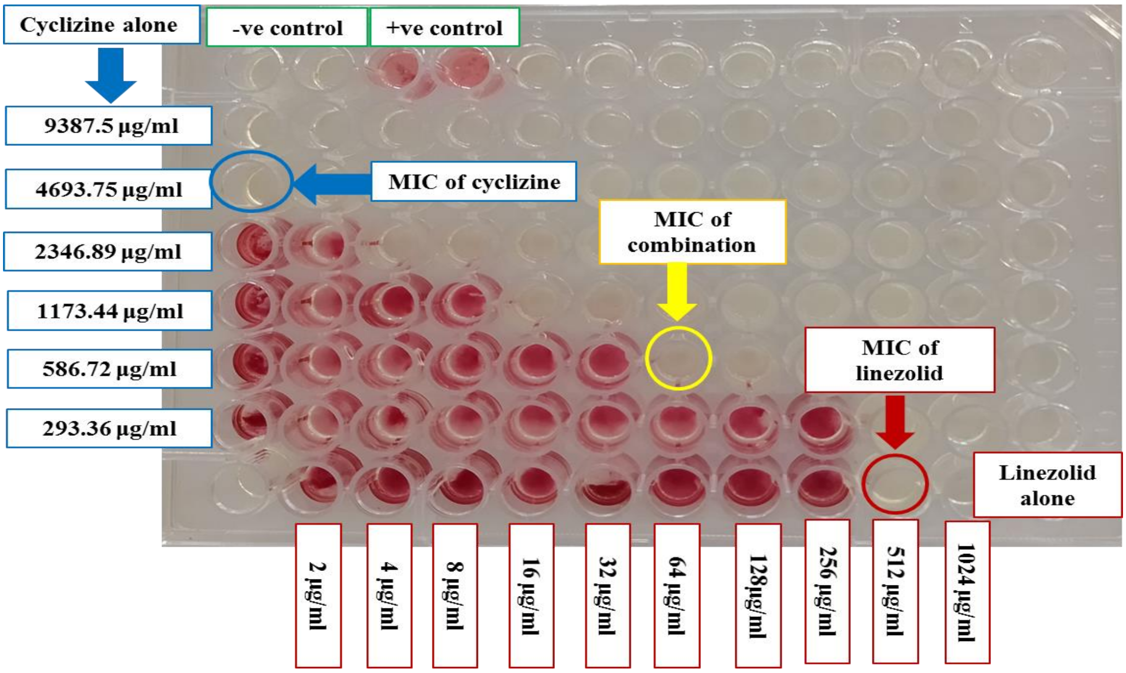

Supplementary Fig. 3: Checkerboard assay for study the inhibitory effect of cyclizine on linezolid resistance. FICI= 0.25 against *S. aureus* isolate No. 40. Bacterial growth was visualized using TTC solution (100 µg/mL). MIC: minimum inhibitory concentration.

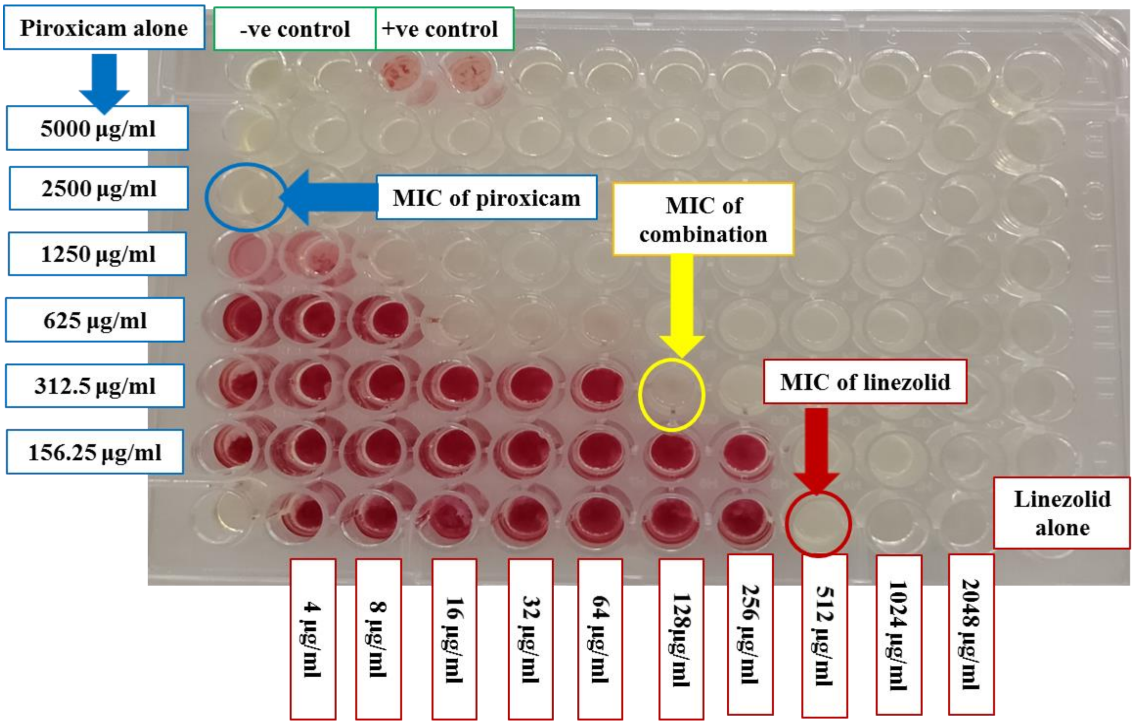

Supplementary Fig. 4: Checkerboard assay for study the inhibitory effect of piroxicam on linezolid resistance. FICI= 0.38 against *S. aureus* isolate No. 40. Bacterial growth was visualized using TTC solution (100 µg/mL). MIC: minimum inhibitory concentration.
